# Non-serous ovarian cancer in PTEN Hamartoma Tumor Syndrome: additional evidence for increased risk

**DOI:** 10.1007/s10689-025-00453-z

**Published:** 2025-03-18

**Authors:** Ane J. Schei-Andersen, Vera M. Witjes, Janet R. Vos, Arjen R. Mensenkamp, Anne van Altena, Jolanda Schieving, Michiel Simons, Janneke H. M. Schuurs-Hoeijmakers, PTEN Study Group, Muriel A. Adank, Liselotte P. van Hest, Yvette van Ierland, Mirjam de Jong, Marjolijn C. J. Jongmans, Edward M. Leter, Maartje Nielsen, Nicoline Hoogerbrugge

**Affiliations:** 1 Department of Human Genetics, Radboud University Medical Center, Nijmegen, The Netherlands; 2 Radboud Institute of Medical Innovation, Radboud University Medical Center, Nijmegen, The Netherlands; 3 Department of Obstetrics and Gynecology, Radboud University Medical Center, Nijmegen, The Netherlands; 4 Department of Pediatric Neurology, Radboud University Medical Center, Amalia Children’s Hospital, Nijmegen, The Netherlands; 5 Department of Pathology, Radboud University Medical Center, Nijmegen, The Netherlands; 6 European Reference Network Genetic Tumor Risk Syndromes (ERN GENTURIS), Nijmegen, The Netherlands

**Keywords:** PTEN, PHTS, Ovarian cancer, Hereditary cancer, Phenotype, Genetics

## Abstract

Increased hereditary cancer risk is one of the hallmarks of PTEN Hamartoma Tumor Syndrome (PHTS) which is caused by a pathogenic germline variant in *PTEN*. Case reports and some cohort studies have described ovarian cancer (OC) in PHTS patients. Previously, we observed an enrichment of non-serous OC in PHTS compared to sporadic cases (3% vs 1%). However, ovarian cancer is currently not considered a PHTS-related cancer. The aim of this study was to describe five PHTS patients with a pathogenic germline variant in *PTEN* with non-serous OC. Three of the non-serous OCs were mucinous carcinomas (49, 51 and 52 years) and two were malignant germ cell tumors (8 and 15 years) and all were diagnosed before genetic testing and PHTS diagnosis. In addition to OC, the described patients developed other PHTS-related benign and malignant lesions. We provide further evidence that non-serous ovarian cancer, especially mucinous, endometrioid and malignant germ cell tumors should be further investigated as potential PHTS-related cancers.

## Introduction

The genetic tumor risk syndrome PTEN Hamartoma Tumor Syndrome (PHTS) is caused by pathogenic germline variants in *PTEN*. PHTS is characterized by multi-systemic overgrowth, including macrocephaly, mucocutaneous lesions, vascular malformations, gastrointestinal lesions, Lhermitte-Duclos disease and benign hamartomatous tumors [1]. Additionally, PHTS patients have an increased risk of developing breast cancer, thyroid cancer, endometrial cancer, colorectal cancer, renal cancer and melanoma [1]. To prevent the development of advanced cancer, PHTS patients are offered cancer surveillance and/or preventive surgery. Ovarian cancer is currently not considered a PHTS-related cancer, though it has been reported in these patients [1].

Ovarian cancer (OC) is diagnosed in approximately 1% of females [2]. The majority of malignant ovarian tumors (> 90%) are epithelial carcinomas, 5–6% are sex-cord stromal tumors and 1–2% are germ cell tumors [3]. In the general population, high-grade serous OC is the predominant group of epithelial OC (> 70%) and is associated with hereditary breast and ovarian cancer due to pathogenic germline variants in *BRCA1/2, PALB2, BRIP1, RAD51C or RAD51D* [2]. While serous OC is the most frequently occurring OC subtype, previous case reports and cohort studies have described the presence of non-serous OC in PHTS patients [4–13]. More specifically, this includes epithelial carcinomas such as endometrioid, clear cell, mucinous, sex-cord stromal tumors and germ cell tumors. Case reports suggest an association and three cohort studies suggest an enrichment of non-serous OC in PHTS patients (Table 1) [4–13].

**Table 1:** Reports and studies on ovarian tumors in PHTS patients

Reference	Country	OC- age	OC – histology	Personal medical history	PTEN variant
Yauy, 2019	France	28	Clear cell	None	Exon 5, c.388C > T, p.(Arg130*)
Matsubayashi, 2019	Japan	31 and 39	Endometrioid (metachronous)	Mammary fibroma, esophageal and skin papilloma, colonic hamartoma, uterine myoma, lipoma	Exon 8, c.1026 + 1G > T
Higuchi, 2022	Japan	55	Carcinosarcoma	Breast cancer, thyroid goiter, palatal papillomatosis	Unknown
Vasovcak, 2011	Czechia	43	Adenopapilocarcinoma	Multiple primary malignancies and subtle mucocutaneous lesions such as small polyps and wart-like papules. Over a period of 23 years, she developed various malignant neoplasms including thyroid, ovarian, stomach, and colon carcinomas, and a benign meningioma	Exon 5, c.438delT, p.Leu146X
Mee-Yon Cho, 2008	Korea	7	Dysgerminoma	Hamartomatous soft tissue extremity mass, diffuse gastrointestinal hamartomatous polyposis, diffuse esophageal glycogenic acanthosis, skin manifestations, papillary thyroid carcinoma and benign follicular thyroid nodules	Exon 7, c.655C > T, p.Gln219*
Kouzuki, 2021	Japan	3	Immature teratoma	Macrocephaly, cavernous hemangioma of forearm, lipoma of chest wall, delayed motor development	Exon 2, c.86delA (p.Tyr29fs)
Habacht, 2021	Austria	4	mixed germ cell tumor with dysgerminoma and mature teratoma components, as well as structures suspicious for a hemangioma component	Lipomas, keratosis pilaris, macrocephaly	Exon 5, c.406 T > C
Smpokou, 2014	USA	16	Granulosa cell tumor	Unknown	Unknown
Schei-Andersen, 2024 (Cohort study)	Netherlands	49 (15—51)	**N = 190, 3%** Five patients: 2 mucinous, 2 germ cell tumors, 1 endometrioid	Unknown	Unknown
Bubien, 2013 (Cohort study)	France	NA	**N = 140, 3%** Four patients: 3 germ cell tumors	Unknown	Unknown
Mighell, 2020 (Cohort study)	USA, gnomAD	NA	**N = 193, 4%** Eight patients: 3 Endometrioid, 3 Not otherwise specified, 1 germ cell tumor, 1 serous	Unknown	Unknown

In a previous study, we observed an enrichment of OC (3%) in our PHTS cohort (341 patients, 190 females) [13]. Given the rarity of non-serous OC in the general female population, multiple observed cases of non-serous OC in PHTS could be indicative of these subtypes being PHTS-related cancers [2]. The aim of this paper is to describe five PHTS patients with non-serous ovarian cancer, their histology, age of diagnosis and other PTHS-related lesions along with an overview of previous literature.

## Methods

Four of the included patients in this case series are sourced from a previous cohort study [13]. We further identified another PHTS patient with non-serous OC in our expertise center. The included patients underwent targeted *PTEN* testing between 1997–2020 in line with testing guidelines at the time of testing at our PHTS expertise center at Radboudumc. The variants were re-classified following the ClinGen PTEN Variant Curation Expert panel’s Variant Interpretation Guidelines for PTEN, Version 3.1.0 [14]. Cancer characteristics and PHTS-related benign lesion data were collected from the Dutch nationwide pathology databank (Palga). A complete staging of the tumors was not possible due to incomplete data. Tumor tissue was not sequenced as samples were old and there are no known *PTEN* mutational signatures in OC. Last follow-up was defined as year of pathology report request (2020). This study was conducted in line with the medical ethics committee guidelines of the Radboudumc. Patient characteristics are presented in Table 2.

**Table 2:** Patient characteristics

	Case 1	Case 2	Case 3	Case 4	Case 5
*PTEN* variant (GRCh37)	c.389G > T, p.(Arg130Leu)	c.165-1G > A, p.(?)	Exon 6 deletion	c.382A > G, p.(Lys128Glu)	c.510 T > G, p.(Ser170Arg)
Ovarian cancer type—Age of diagnosis	Germ cell tumor – 8 years old	Germ cell tumor – 15 years old	Mucinous adenocarcinoma – 49 years old	Mucinous adenocarcinoma – 51 years old	Mucinous adenocarcinoma – 52 years old
Other cancer diagnoses—Age of diagnosis	Not present	Breast cancer – Infiltrating Ductal Carcinoma – 37 years old	Thyroid cancer – Follicular carcinoma – 25 years old	Breast cancer—Infiltrating Ductal Carcinoma – 48 years old Skin cancer—Squamous Cell carcinoma – 56 years old	Breast cancer—Phyllodes tumor – 51 years old and Infiltrating Ductal adenocarcinoma – 53 years old Skin cancer – Basal cell carcinoma – 54 years old
Benign lesions—Age of diagnosis	Thyroid: Hyperplastic nodules – 11, 13, 14 & Goiter – 16 years old	Skin: Fibroma – 27 years old Vascular: Hemangiomas – 22, 23, 33 & 39 years old Breast: Fibrocystic breast disease & fibromas – 37 & 38 years old Uterus: Endometrial Hyperplasia – 40 years old Gastrointestinal: hyperplastic polyp, ganglioneuroma, tubular adenoma – 40 & 43 years old	Skin: Fibroma – 32 & 63 years old, Papillomas – 38 & 39 years old	Breast: Fibrocystic breast disease – 30 & 52 years old Thyroid: Goiter & Follicular adenoma – 36 years old Uterus: Endometrial Hyperplasia – 40 years old Skin: Fibroma – 44 & 47 years old, Lipoma – 55 years old Gastrointestinal: Hyperplastic polyp, inflammatory polyp, sessile serrated lesion, tubular adenomas – 48–54 years old Vascular: Hemangioma – 53 years old	Thyroid: Goiter – 31 years old Vascular: Hemangiomas – 32, 36 & 46 years old Skin: Lipoma – 42 years old Gastrointestinal: Hyperplastic polyps, sessile serrated lesion, tubular adenoma – 58 & 59 years old

## Clinical reports

### Case 1

A malignant germ cell tumor of unknown stage was removed from the right ovary of an 8 year old patient. The tumor was surgically removed along with mesorectal tissue. The tumor was large (17 × 10x6cm, 429 g) and characterized by necrotic tissue, fibrohystiocytic clearance, chronic inflammation and calcification.

The patient underwent germline testing 2 years later at 10 years of age which revealed a likely pathogenic germline *PTEN* missense variant in exon 5 (NM_000314.8: c.389G > T, p.(Arg130Leu)). In the following years, multiple tissue biopsies of the thyroid showed multinodular hyperplasia at ages 11, 13 and 14 and the patient underwent complete thyroidectomy at age 16. The patient had no other biopsies or excisions and was 27 years old at last follow-up.

### Case 2

This patient was diagnosed with a malignant mixed germ cell tumor of the right ovary with pT1aNxMx (FIGO stage 1a) at 15 years of age. The tumor was of mixed type including dysgerminoma, embryonal cell carcinoma and yolk sac tumor.

This patient had *PTEN* testing years later at age 35 due to personal medical history (keratotic pits of the palm, oral papillomas, multinodular goiter and macrocephaly) and presented with a pathogenic germline *PTEN* single nucleotide substitution in intron 2 affecting the splice site (NM_000314.8: c.165-1G > A, p.(?)).

Twenty-two years after the ovarian cancer diagnosis, at age 37, the patient was diagnosed with an infiltrating ductal carcinoma NOS in the upper outer quadrant of the left breast at grade 2 with pT1cN0Mx (stage 1a). There were three invasive tumors along with a smaller DCIS component (grade 3 with microcalcification and comedonecrosis). The tumors were estrogen (ER) and progesterone (PR) receptor positive and human epidermal growth factor 2 receptor (Her2Neu) negative. Benign breast lesions, including fibroadenomas, microcysts, ductal hyperplasia and apocrine metaplasia were also observed. The patient underwent bilateral mastectomy.

This patient has throughout life presented with other PHTS-related lesions, including hyperplastic polyps, ganglioneuromas and tubular adenomas of the colon at 40 and 43 years old, fibroma of the skin (27 years old), multiple hemangiomas (22, 32, 33 and 39 years old) and endometrial hyperplasia at 40 years of age. The patient was 44 years old at last of follow-up.

### Case 3

This 49 year-old patient presented with a mucinous adenocarcinoma of unknown stage which was treated with systemic chemotherapy and salpingo-oophorectomy.

This patient underwent germline *PTEN* testing at 57 years of age following suspicion of PHTS (macrocephaly, gingival hypertrophy and oral papillomas) which revealed a large likely pathogenic germline *PTEN* deletion covering the entirety of exon 6.

Ovarian cancer was the patient’s second malignancy as she had previously been diagnosed with thyroid cancer at 25 years old, consisting of a follicular carcinoma at grade 1 and unknown stage.

This patient also presented with other PHTS-related lesions, including inflammatory, hyperplastic, hamartomatous polyps, ganglioneuromas and tubular adenomas of the colon at 58 years old and fibromas (32 and 63 years old) and papillomas (38 and 39 years old) of the skin. This patient passed away at 65 years of age of unknown cause.

### Case 4

This 51 year old patient presented with a T1aNxMx (FIGO stage 1a) mucinous carcinoma of the right ovary, measuring 35.5 cm with multicystic components. The patient underwent an ovariectomy of the right ovary.

The patient underwent germline *PTEN* testing on suspicion of PHTS (goiter, macrocephaly and breast cancer) at age 52 which revealed a pathogenic germline *PTEN* missense variant in exon 5 (NM_000314.8: c.382A > G, p.(Lys128Glu)).

This patient was diagnosed with breast cancer at 48 years old, concerning a metastatic infiltrating ductal carcinoma NOS of the left breast at grade 2, pT3N1Mx (stage 3a) classification ER, PR positive and Her2Neu negative with high-grade DCIS. The patient underwent mastectomy of the left breast after neoadjuvant chemotherapy.

She was diagnosed with skin cancer on the left side of the scalp at age 56, a squamous cell carcinoma grade 1 with pT1NxMx (stage 1) treated by excision of the affected skin.

This patient presented with other PHTS-related lesions, including multinodular goiter and follicular adenoma at 36 years old treated by total thyroidectomy and endometrial hyperplasia at 40 years old treated by hysterectomy. She also presented with multiple hyperplastic, inflammatory polyps, sessile serrated lesions and tubular adenomas of the colon between the ages 48 and 54, bilateral fibrocystic breast disease at 30 and 53 years of age, fibromas (44 and 47 years old) and lipoma (55 years) of the skin and hemangioma (53 years). The patient was 59 years old at last follow-up.

### Case 5

A 52 year old patient presented with mucinous ovarian carcinoma of the left ovary grade 1 with pT1aNxMx (FIGO stage 1a). The patient underwent a hysterectomy and salpingo-oophorectomy.

This patient underwent genetic testing at 59 years old due to cascade testing which revealed a pathogenic germline *PTEN* missense variant in exon 6 (NM_000314.8: c.510 T > G, p.(Ser170Arg)).

The patient also developed bilateral breast cancer and skin cancer. The first breast cancer occurred at 51 years old in the left breast and was a malignant Phyllodes tumor with pT2NxMx (stage 2a) and DCIS grade 1. The second breast cancer developed at 53 years old in the right breast and was an infiltrating ductal adenocarcinoma at grade 1 with pT1bN0Mx (stage 1a) and a low grade DCIS component. The tumors were ER and PR positive and Her2Neu negative. Both breast cancers were treated by lumpectomy. The patient developed skin cancer at 54 years old, a basal cell carcinoma of the forehead with unknown stage, which was removed by excision.

This patient developed other PHTS-related lesions, including thyroid goiter at 31 years old treated by total thyroidectomy, hemangiomas at age 32, 36 and 46, lipoma of the shoulder at 42 years old and gastrointestinal lesions, including sessile serrated lesions, hyperplasic polyps and tubular adenomas at 58 and 59 years old. The patient was 63 years old at last follow-up.

## Discussion

Here, we presented five PHTS patients with pathology-confirmed non-serous ovarian cancer. All patients developed non-serous OC before germline *PTEN* testing. Additionally, all patients developed other malignant or benign PHTS-related lesions. Three patients developed mucinous OC (49, 51 and 52 years) and two germ cell tumors (8 and 15 years) at similar ages as the general population where mucinous OC occurs at a mean age of 45 years whereas germ cell tumors are most frequent between 15 and 19 years old [15, 16]. Two of the presented cases and 3 cases from literature had pathogenic germline variants in Exon 5 of *PTEN,* further highlighting this exon as a mutational hot spot.

Some OCs are of hereditary origin where pathogenic germline variants in *BRCA1/2* accounting for 10–15% of OC cases and together with *RAD51C*, *RAD51D*, *BRIP1* and *PALB2,* account for 36% of high-grade serous OC [2]. Patients with Lynch syndrome have a lifetime risk up to 20% and develop most commonly endometrioid or clear cell OC [2]. Up to 10% of female patients with *DICER1* tumor predisposition may develop sex cord-stromal tumors and approximately 50% of patients with Sertoli-Leydig tumors and gynandroblastomas have a pathogenic germline *DICER1* variant [17]. Germ cell tumors have been observed in Activated PI3-kinase Delta Syndrome (APDS) [18]. APDS is, like *PTEN*, modulated by the PI3K pathway which may indicate an association between the pathway and germ cell tumor development.

OC is currently not considered a part of the PHTS phenotype. However, case reports suggest an association and three cohort studies suggest an enrichment of OC in PHTS [4–9, 13, 19]. Interestingly, the majority consists of non-serous histologies whereas in the general population high-grade serous is most prevalent [2]. TP53 loss and BRCA1/2 alterations are characteristic and presumed initiating events in carcinogenesis in high-grade serous ovarian cancer, whilst PI3K/AKT/PTEN pathway hyperactivation is presumed to play a role in part of endometrial ovarian tumors and in the development of germ cell tumors [20–22]. This might explain the enrichment we observed for non-serous OC although further molecular studies are needed. Additionally, the study by Cummings et al. on cancer risk in PHTS (N = 193) revealed an age-, family history- and ancestry-adjusted increased odds ratio (3.77) for OC, further emphasizing a possible increased risk of OC in PHTS [23]. The patients in this case series underwent *PTEN* testing years after OC diagnosis as clinicians did not suspect PHTS based on the OC diagnosis. Consequently, patients (and their families) may not be referred for *PTEN* testing and do not receive appropriate care. Therefore, it is important to consider the possibility that non-serous OC may be a PHTS-related cancer and PHTS might be included in the differential diagnosis of non-serous OC. There is currently not enough data to support surveillance or preventive treatment for non-serous OC in PHTS patients and further research is needed such as tumor sequencing to assess the role of PTEN in pathogenesis of non-serous OC in PHTS patients along with further studies to establish lifetime risks and usefulness of surveillance.

We presented five PHTS patients with non-serous ovarian cancer, their PHTS-related features and genetic variants. Taken together with previous reports and studies, we provide further evidence that non-serous ovarian cancer, specifically mucinous, endometrioid and malignant germ cell tumors, may be PHTS-related cancers. Further studies should investigate this association.

## Data Availability

No datasets were generated or analysed during the current study.
